# “*No forest, no future, but they don’t see us*”: eco-anxiety, inequality, and environmental injustice in São Paulo

**DOI:** 10.3389/fpubh.2025.1555386

**Published:** 2025-06-05

**Authors:** Marcia Thereza Couto, Marina Senent-Valero, Lorruan Alves dos Santos, Milena Mateuzi Carmo, Alicia Matijasevich, Maria Pastor-Valero

**Affiliations:** ^1^Departamento de Medicina Preventiva, Faculdade de Medicina FMUSP, Universidade de São Paulo, São Paulo, Brazil; ^2^Departamento de Salud Pública, Historia de la Ciencia y Ginecología, Facultad de Medicina, Universidad Miguel Hernández, Alicante, Spain; ^3^Servicio de Dermatología, Hospital General Universitario Dr. Balmis, Instituto de Investigación Sanitaria y Biomédica de Alicante (ISABIAL), Alicante, Spain; ^4^CIBER in Epidemiology and Public Health (CIBERESP), Madrid, Spain; ^5^Programa de Pós-Graduação em Saúde Coletiva, Faculdade de Medicina FMUSP, Universidade de São Paulo, São Paulo, Brazil

**Keywords:** eco-anxiety, climate change health impact, climate change adaptation, environmental injustice, Global South

## Abstract

**Introduction:**

Eco-anxiety disproportionately affects vulnerable populations and younger generations. High and chronic levels of eco-anxiety may have significant impacts on mental health.

**Methods:**

This qualitative study, utilizing a guided group discussion methodology, aimed to explore perceptions of climate change, eco-anxiety, health impacts, resilience, pro-environmental behaviors, and opinions on governmental and institutional actions in São Paulo, Brazil. Six focus groups were conducted: four with young residents and women community leaders from marginalized communities, and two with university students from higher-socioeconomic backgrounds.

**Results:**

Perceptions of climate change varied: more vulnerable participants linked it to personal experiences but lacked understanding of its causes, while higher-income students relied on formal education. Though unfamiliar with “eco-anxiety,” participants expressed related feelings. Vulnerable communities reported greater trauma and health impacts, with Black women leaders emphasizing how climate change exacerbates racial and gender inequalities. Community networks were vital for resilience, but climate change disrupted future plans—vulnerable groups faced immediate losses, while higher-income students made long-term choices like delaying parenthood. Poverty hindered collective pro environmental behavior in vulnerable communities. Participants from the outskirt criticized environmental messaging for neglecting their heightened risks. Across all groups, government inaction and prioritization of economic interests over environmental policies were key concerns.

**Conclusion:**

Poverty and exclusion drive and exacerbate climate vulnerability, with marginalized populations often feeling their experiences are overlooked in climate discourse. These findings may offer valuable insights into the socio-political dimensions of climate vulnerability in other Global South contexts.

## 1 Introduction

Global warming and climate change (CC) extremes—such as heatwaves, floods, droughts, and typhoons—have intensified in recent years, disproportionately impacting vulnerable populations, younger generations, those dependent on natural resources, and individuals with pre-existing mental and physical health conditions (1). Differential human vulnerability to CC is shaped by a complex interplay of social, economic, historical, political and geographical factors, including racial identities such as Black, Asian, and Indigenous populations. These groups often face greater exposure to climate-related hazards, reduced access to adaptive resources, and increased mental health challenges, all of which could exacerbate their experience of eco-anxiety (2, 3).

Eco-anxiety is an emotional response to CC, characterized by fear, worry, guilt, and hopelessness. While it affects individuals of all ages, younger people, with more years of life ahead, may experience a greater sense of uncertainty and pessimism about their future (4–6). In some cases, this response can be considered an adaptive reaction to CC, referred to as “practical anxiety” (7) and might prompt individuals to reassess their behavior and adopt environmentally sustainable practices (8, 9). In other cases, high and sustained levels of eco-anxiety can cause a deterioration of mental health, including symptoms of depression, anxiety, stress, insomnia, lower self-reported mental health and functional impairment (3). Gender may also be associated with increased vulnerability to climate anxiety (CA), in part due to factors related to gender roles and unequal access to power, information, and financial resources (6, 10, 11). Young people are highly vulnerable to eco-anxiety due to their frequent internet use, which amplifies exposure to constant media coverage of environmental threats (11). This increased exposure, combined with a heightened sensitivity to feelings of government betrayal and inaction, can act as chronic stressors, further exacerbating their mental health challenges and negatively impacting their daily functioning (6).

Several studies have shown that direct experience of CC is associated with greater CA around the world (7, 10, 12). However, the literature indicates significant variability in CA levels across countries in the Global North and Global South (13). The relationship between CC, CA, and other relevant contextual factors remains complex and is not yet fully understood.

As the acute and long-term effects of CC continue to expand, the impacts on both physical and mental health will intensify, with the most severe consequences affecting vulnerable populations (5, 8, 14). To date, mainstream discourse and research on negative emotional responses to CC have mainly focused on the experiences and perspectives of White, Western populations (15).

This qualitative study carried out in São Paulo, Brazil, aimed to explore how CC affects health, emotions, and lived experiences among marginalized populations from communities on the city’s outskirts, including youth and women community leaders, as well as middle- to high-income university students. Additionally, the study examines pro-environmental behaviors, resilience strategies, and perceptions of government and institutional roles in CC mitigation.

## 2 Materials and methods

### 2.1 Design of the study

This qualitative study uses focus groups (FGs) to thoroughly explore key themes, promoting interaction and discussion among participants. This approach facilitates the emergence of insights, idea development and contrasting viewpoints (16). The study follows the principles of qualitative research and used the COnsolidated criteria for REporting Qualitative research (COREQ) (17).

### 2.2 Scope of the study, participants, and recruitment

The study was conducted in São Paulo, Brazil’s most populous city, with approximately 11.5 million residents. The city is characterized by significant inequality, manifesting itself across race, class, gender and space. Politically induced territorial expansion since the 1960s and 1970s has seen investment concentrated in wealthier central areas, neglecting peripheral zones where many migrants from rural regions live in inadequate conditions (18, 19). Consequently, the peripheries house a predominantly poor, Black and young population, while central regions are inhabited by better-off, largely White families. Although grassroots social movements have improved access to fundamental rights and urban infrastructure over the past five decades (20, 21), this remains insufficient, particularly in housing and healthcare. Moreover, growing property speculation is pushing marginalized residents into riskier areas characterized by precarious self-constructed housing.

**Figure 1:**
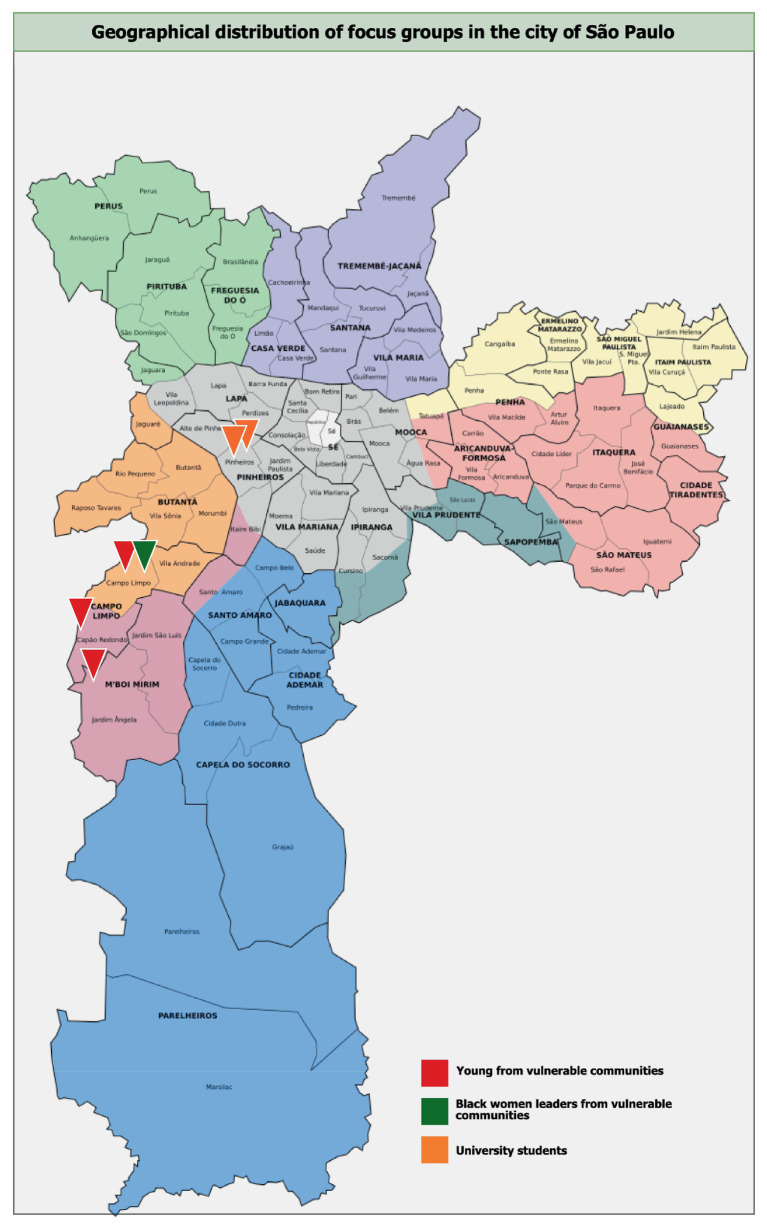
Groups 1, 3, and 5: Young people from vulnerable communities, districts of Jardim Ângela, Campo Limpo and Capão Redondo (South Zone), respectively. Group 2: Black women leaders from vulnerable communities, district of Campo Limpo (South Zone). Groups 4 and 6: University students (medicine, history, psychology and geography), district of Pinheiros (West Zone).

Participants were recruited using purposive sampling and the snowball technique. Local leaders from marginalized communities on the outskirts of São Paulo invited young and women community leaders, ensuring diversity in age, gender, and race/ethnicity. Similarly, student representatives contacted university students from Universidade de São Paulo (USP). The study population comprised six focus groups: three with young residents from vulnerable communities on the outskirts of São Paulo (groups 1, 3, and 5), two with university students from middle- to high-income backgrounds (groups 4 and 6), and one with women leaders from vulnerable communities, primarily involved in feminism and the Black women’s movement (group 2). Participants had to meet the following inclusion criteria: be residents of the city of São Paulo, be aged 18 to 30 for the younger groups and 25 to 60 for the group of women leaders. We included the latter group for their first-hand knowledge of community needs and their longer experience, as they were older than the other groups, to reflect on the progression of CC and its impact (Figure 1, describes the geographical location of each of the FGs). Participants had to meet the following inclusion criteria: be residents of the city of São Paulo, be aged 18 to 30 for the younger groups and 25 to 60 for the group of women leaders. Recruitment was done via purposive sampling through local leaders, ensuring diversity in age, gender and race/ethnicity.

### 2.3 Conceptual framework

The study coordinators constructed a conceptual framework to examine the phenomenon of eco-anxiety from a public health perspective. This framework was developed based on a systematic review previously conducted by our team, which critically assessed the existing evidence on eco-anxiety and its health implications (3). It was further expanded by incorporating findings from studies addressing related themes—such as resilience, pro-environmental behaviors, and the roles of governments and institutions in responding to CC—as well as expert input (see Supplementary material, Annex A). Drawing on this foundation, we designed a FG script to explore a wide range of interconnected topics, including eco-anxiety, participants’ perceptions, health impacts, resilience strategies, support networks, pro-environmental behaviors, and perceived responsibilities of governments and institutions in addressing CC (see Supplementary material, Annex B).

### 2.4 Data collection

Three interviewers with expertise in qualitative research methods conducted the FGs from December 6, 2022, to February 10, 2023, with each FG lasting from 80 to 90 min. Each session was audio recorded, transcribed, anonymized and categorized using MAXQDA software (version 2022). After obtaining informed consent, the participants anonymously completed a self-reported sociodemographic questionnaire (Supplementary material, Annex C). At the start of the FGs, participants selected one of 10 images illustrating CC scenarios to convey their understanding, feelings and experiences. The images were sourced from online indexing sites (Supplementary material, Annex D).

### 2.5 Analytical procedure

The data were analyzed and interpreted using thematic analysis (22), focusing on the content and contextual meaning of the narratives. The inductive analytical-interpretative process included: (a) comprehensive reading for immersion and understanding of the focus group transcripts; (b) identification of emerging themes from the discussions; (c) recognition of explicit and implicit patterns of meaning by three researchers, including the facilitator and two verification specialists; (d) exploration of broader sociocultural meanings underlying participants’ statements; (e) dialogue between discussed ideas and comparisons with existing literature; and (f) collaborative development of an interpretative synthesis among researchers, aligned with the study’s objectives, findings and relevant discussions from the literature. The analysis was performed independently and triangulated between the interviewers and another member of the research team, followed by discussion and consensus as a procedure for validation and quality control of the results obtained.

### 2.6 Ethical aspects

The study was reviewed by the Ethics Committee for the Analysis of Research Projects (CAPPesq) of the Hospital de Clínicas at the University of São Paulo, with approval number [63469522.6.0000.0068]. All participants were informed of the study’s objectives and signed the informed consent form prior to the collection of sociodemographic data on individual forms and the conduct of the FGs. Their participation was voluntary and anonymous, and they understood that they could withdraw from the study at any time without explanation. During the FGs, the comfort of the participants was a priority at all times, as was confidentiality.

### 2.7 Rigor

The results of the analysis were presented to the participants, obtaining their agreement, with both parties deciding to remove identifiable information from the verbatim quotes that feature in this article.

## 3 Results

Table 1 presents the self-reported sociodemographic and health characteristics by the 62 participants. Each focused group consisted of 9 to 12 participants: 3 groups of suburban youth (Groups 1, 3, 5; *n* = 29), 2 groups of university students (Groups 4, 6; *n* = 22) and 1 group of Black women community leaders (Group 2; *n* = 11). In sum, the mean age was 23.5 years (range: 18–59), with most identifying as cisgender women (61%) and cisgender men (35%). Most of the participants identified as Black or Brown-skinned (48%), followed by White (39%), Asian (7%) and Indigenous populations of the Americas (3%). All the women in the group of leaders declared themselves to be Black.

**Table 1:** Participants ´sociodemographic and health characteristics (*n* = 62).

Variables	Total *n* (%)	Women community leaders	Young people from vulnerable community	University students
		*N* = 11	*N* = 29	*N* = 22
Sociodemographic characteristics				
Age (mean)	**23.5 (18–59)**	39.7 (26–59)	19.7 (18–28)	20 (18–24)
Identity gender				
Cis-gender woman	**38 (61)**	9	17	12
Cis-gender man	**22 (35)**	2	11	9
Transgender man	**1 (2)**	0	1	0
Non-binary person	**1 (2)**	0	0	1
Sexual orientation				
Heterosexual	**36 (58)**	7	15	14
Bisexual	**17 (27)**	2	9	6
Homosexual	**3 (5)**	1	1	1
Pansexual	**3 (5)**	0	3	0
Lesbian	**2 (3)**	1	0	1
Other	**1 (2)**	0	1	0
^*^Race/skin color				
White	**24 (39)**	1	7	16
Black	**18 (29)**	5	13	0
Brown	**12 (19)**	5	6	1
Asian	**4 (7)**	0	0	4
Indigenous	**2 (3)**	0	2	0
Other	**2 (3)**	0	1	1
Educational level				
Tertiary education	**9 (15)**	5	3	1
Incomplete tertiary education	**28 (45)**	3	4	21
Secondary education	**22 (35)**	1	21	0
Primary (or lower) education	**3 (5)**	2	1	0
Employment				
No	**37 (59)**	2	15	20
Yes	**24 (39)**	9	13	2
NR	**1 (2)**	0	1	0
Health status				
Current physical disease				
No	**50 (81)**	8	25	17
Yes	**12 (19)**	3	4	5
Current physical disease				
Rhinitis	**3 (5)**	0	2	1
Hypertension	**2 (3)**	2	0	0
Migraine	**2 (3)**	0	0	2
Hypothyroidism	**1 (2)**	0	1	0
DM	**1 (2)**	1	0	0
Asthma	**1 (2)**	0	1	0
Bone spurs	**1 (2)**	1	0	0
Weakness	**1 (2)**	1	0	0
Endometriosis	**1 (2)**	0	0	1
Patellofemoral impingement	**1 (2)**	0	0	1
Use of chronic treatment				
No	**54 (87)**	8	27	19
Yes	**8 (13)**	3	2	3
Diagnosis of a mental health disorder				
No	**24 (39)**	6	10	8
Yes	**38 (61)**	5	19	14
Regular use of mental health medication				
No	**54 (87)**	11	25	18
Yes	**8 (13)**	0	4	4
Mental health disorders				
Anxiety	**19 (31)**	3	9	7
Depression	**7 (11)**	1	3	3
Panic attacks	**4 (7)**	3	0	1
BPD	**2 (3)**	0	1	1
ASD	**1 (2)**	1	0	0
PTSD	**1 (2)**	1	0	0
ADHD	**1 (2)**	0	1	0
Bulimia	**1 (2)**	0	1	0
Traumatic distress	**1 (2)**	1	0	0
OCD	**1 (2)**	0	0	1
Being sad/depressed most days during the last month				
No	**34 (55)**	4	12	18
Yes	**28 (45)**	7	17	4

*Race/skin color as defined by the Census, IBGE (Brazilian Institute of Geography and Statistics).

BPD, Borderline personality disorder; ASD, Acute stress disorder; DM, Diabetes Mellitus; PTSD, Post-traumatic stress disorder; ADHD, Attention deficit hyperactivity disorder; OCD, Obsessive compulsive disorder; NR, Non-reported.Bold values reflect the data for the whole population.

The majority, 28 (45%) had incomplete tertiary education (less than 12 years) followed by 22 (35%) who had secondary education, 9 (15%) had tertiary education (undergraduate and graduate), and 3 (5%) had primary or lower education (2 of them with a duration of 5 years and 1 of them from 6 to 8 years). Almost 59% were unemployed, and 19% reported having chronic diseases, primarily allergic rhinitis (5%), migraine (3%) and hypertension (3%). Additionally, 61% had a clinical diagnosis of a mental disorder, predominantly anxiety (31%); 45% reported feeling sad or depressed most days in the preceding month. Only 13% were undergoing medical therapy. Further detailed information about sociodemographic and health characteristics of the participants is provided in Supplementary material, Annex E.

The results of the FGs were organized into nine thematic categories, main insights from all FG are described below.

### 3.1 Perceptions (knowledge, belief) about climate change

Most of the participants, regardless of their age or where they lived were aware of CC, associating it primarily with rising temperatures, along with floods, landslides, fires, pollution, and unpredictable weather patterns. Among vulnerable populations, the perception of CC stemmed from direct experiences with its effects, while the understanding of university students was shaped by information on more distant impacts, often acquired in an educational context:

Climate change is affecting our lives, you know? It gets up to 40 degrees, then suddenly the temperature drops to 27, 26. We get rain, sun, storms—all the seasons in one day… it’s getting more and more out of control. Group 3 - Young people from the periphery

I think about it in a more historical sense. Thinking about the industrial revolution, all the research shows that from 1800 until now is when the world’s temperature has increased the most. Group 4 - Young university students

Some participants from vulnerable communities stated that they did not know what CC was or its causes, to the point of not identifying it as something close to their reality:

People don’t know what climate change is, how the climate works, how the weather works. Group 2 - Women community leaders

I think climate change is a television thing. It’s as if I’m not part of it. It’s a distant thing, newspaper news. Group 2 - Women community leaders

### 3.2 Feelings and emotions related to climate change

The vulnerable population expressed constant feelings of sadness, fear, uncertainty, hopelessness, concern, anger and helplessness in the face of the impact of the CC:

It’s very sad, the people in the community struggle a lot to get things and then suddenly a heavy rain comes and takes everything away, many lose their homes and go [to live] on the street. Group 5 - Young people from the periphery

When I see a house sliding downhill, Mums working away, children at home on their own. It’s a business that we can’t predict… And it’s going to swallow up my children, they’re going to die there, buried… I’m not even at home to think about running with them or putting my body on top of them so that the earth falls on me, but it doesn’t fall on them. This kind of thing consumes us… because it becomes a constant worry, because this is not a situation that we’re going to change, we’re not going to be able to change this risky area overnight, every year there are hundreds of deaths and nothing changes. Group 2 - Women community leaders

Young university students also expressed anger about the impact of CC on more vulnerable populations, often distant from their own reality.

We’re very angry here, in this room, in an upscale neighbourhood, in a university, which, although it’s public, is very elitist… because the effect of climate change is far removed from our reality and has an effect on the most vulnerable populations, people living on the streets. If we go out into the corridor, there will be someone cleaning this room who is affected by intense climate change events and who does not have much chance of overcoming these problems. Group 4 - Young university students

Some university students expressed feelings of frustration and resignation as they felt overwhelmed by the difficulties generated by the CC.

Forget about it, let it go, because either we turn this anguish into real action, make this thing change, or we’ll freak out. If you think about it and do not do anything, you’ll go crazy… but I think it’s much easier for us… let go and forget. Group 6 - Young university students

### 3.3 Concrete experiences and perceptions associated with climate change

Participants living in vulnerable communities reported having suffered, either themselves or family members or neighbors, more frequently from violent CC events involving loss of life and property.

Imagine losing your Mum because of climate issues [living in a high-risk area for landslides]. It’s horrible, and it’s even worse because I can’t afford to buy a house. I don’t have a job, I have no income, I have nothing. Group 1 - Young people from the periphery

(participant weeps) I lost an uncle, he had just arrived from Bahia… he was putting up a shack to settle in while he tried to find a permanent place [to live]. It rained, and the hill collapsed, he couldn’t get out… It’s hard when you have to live in a place that isn’t safe, and you have to stay there because you have no other choice. Group 3 - Young people from the periphery

Women leaders in the community demonstrated substantial knowledge about the impacts of CC, linking the appearance of rodents and insects in their homes to increasing temperatures and human activities, such as altering river flows, deforestation and inadequate sanitation infrastructure.

They filled in the river, and this is what we get: poor sanitation, heat, rain. Filling in the river affected the soil, and that’s what causes this: all these animals, rats, cockroaches, coming into people’s homes. Group 2 - Women community leaders

In addition, several women reported an incident of racial and gender-based violence, in which a White man expressed racist attitudes toward a Black woman for using water to wash his garden. Participants emphasized that, while the incident was framed in the context of CC, they perceived it primarily as an act of racism rather than an environmental concern:

The weirdest thing I’ve seen because of climate change? A woman was washing her yard, then a White man walked by, something about climate change, save the animals, and he yanked her hose and hit her hard with it. It was on TV. And he justified that by saying she was destroying the planet [because she was] washing her yard. I really think it was just racism, not anything about [concern for] climate change. He was very White, and she was very Black. Group 2 - Women community leaders

In contrast to these realities, the accounts of the university students did not reveal any extreme circumstances that would compromise their personal safety, or that of their families or the integrity of their homes:

At the beginning of last year it flooded, I tried to take the subway, but everything was at a standstill, [so] I went back home. [I thought] I’m not going to make it, I just can’t go. It was really overwhelming. That’s the only time I remember something truly affecting me. Group 4 - Young university students

### 3.4 Perception of social inequalities and their relationship with climate change

All the groups emphasized the link between social inequalities and CC, viewing disparities as both a cause and a consequence of the impacts of CC. Participants reported having strong thoughts and experiences around environmental injustice, noting that poorer communities suffer disproportionately from CC effects and have less capacity to respond. The university students acknowledged their privilege and safer environments, which shield them from severe CC-related problems.

The ones who really suffer with these problems are us here in the outskirts, the ones marginalized by society. We definitely need to do our part by not littering and doing things the right way. But if you compare how much we pollute to how much the industries, big landowners, and, yeah, mining pollute, there’s no comparison. So we don’t experience it the same way. While we suffer, they’re profiting and smiling. Group 5 - Young people from the periphery

The landslide that affected some of the residents made me relate it to social inequality. Because this place is clearly not safe. It’s not a place to build a house. But these people built their homes here because they had no choice, you know? They didn’t have the money to buy a house in a safer place, in a more expensive place, so they had to live here, on the side of a cliff, on the side of a stream. Many people all over Brazil go through this and also… I also live more or less near a stream and I often see some residents throwing rubbish into the stream, you know? They don’t look after the place properly either, you know? It’s these two things, inequality and the lack of care on the part of the people themselves… that cause some disasters. Group 3 - Young people from the periphery

There is a strong perception of inequality in society’s handling of the consequences of CC, with economic elites neglecting the needs and challenges faced by the most disadvantaged segments of society:

These White middle-class people holding signs that say ‘no forest, no future,’ but they don’t see us. This economic elite, this whiteness that defends the forest but doesn’t defend human beings, doesn’t defend the poor. It’s the same crowd that talks about human rights, [and I ask myself] human rights for who? It’s the same with climate security. Let’s think about it—it should also be a human right. We’re all human beings. The slogan ‘no forest, no future’ isn’t thinking about the poor in the favelas, it isn’t thinking about the kids playing football by the stream. Group 2 - Women community leaders

Young university students recognize the clear relationship between poverty and the impact of CC and how the capacity to respond to adversity is undermined by socioeconomic inequalities:

How are these people supposed to have access to citizenship and decent healthcare? When they open the door, there’s rubbish; their house can’t even have a window that really allows air to circulate… The social aspect of climate change is closely tied to huge inequality… and that makes me really angry at myself. Group 6 - Young university students

This social issue of climate change is related to a very great inequality, which arouses a great deal of anger in me. Group 4 - Young university students

Moreover, they described the intrinsic connection between social inequalities and the impact of CC on the health of the population in the periphery:

I want to talk about a beneficiary I work with. The woman lives in a house where, from street level, you go down a long staircase, and there’s a river behind her house that flows underneath… So the river water and the street water would flood her house. She had the option to move somewhere else, but she didn’t want to because she spent five years trying to get a prosthesis, and she finally managed to get it now. She would say, ‘If I leave here, I’ll lose my allocated health clinic and won’t be able to get the prosthesis done, so I have to stay here. Group 2 - Women community leaders

### 3.5 Climate change and impact on health

All participants associated CC with increased physical and mental ill-health, but the narratives showed a clear difference in the impact of CC on health according to population groups. Residents in peripheral areas reported increased frequency of rhinitis, allergies, headaches, fatigue and infectious diseases such as leptospirosis and mycosis:

There are so many temperature changes in the same day in São Paulo, and my body just can’t keep up with it. I end up getting sicker. I’m always sick… I’m almost never healthy. Group 3 - Young people from the periphery

In addition, they described anxiety and panic attacks, post-traumatic stress disorders (PTSD), and depression, people who are left without strength, plunged into total despondency:

And on the day of that typhoon, the wind was so strong it ripped all the tiles off my house… It was a huge blow for me because my home, which should be a safe place for me, ended up causing me so much despair. [So] now, every time there’s a windstorm, I have a panic attack. Group 1 - Young people from the periphery

And I’ve seen a lot of people… I’ve seen a woman who… couldn’t bring up her children, she couldn’t feed her children… Living through that ended her life. It finished her off. Group 2 - Women community leaders

The university students did not mention having physical illnesses linked to CC, but they talked about anxiety and panic attacks, mostly triggered by hearing about events happening far away. They emphasized using private therapy as a way to cope with these effects:

Yeah, I go to therapy, and that’s something that’s helped me a lot because I used to suffer a lot. I remember when I was little, I would have anxiety attacks when I saw those [climate crisis] images in my geography textbook. I’d start having anxiety attacks, almost a full-blown panic attack. Group 6 - Young university students

### 3.6 Support networks to face severe climate crises

In critical situations caused by CC, participants from vulnerable communities showed resilience and stated that their main support comes from family and community networks:

I’m sure that if any house here floods, because we’re a group that knows each other, from the same community, we’re going to help each other out. Group 2 - Women community leaders

However, people from vulnerable communities reported that family or community support networks are insufficient to address their mental health needs. They also stated that accessing long-term psychological treatment through the Unified Health System (Sistema Único de Saúde—SUS) is very difficult:

For someone who has nothing, they have to wait a long time in the SUS line to see a psychologist once a month. Anyone with mental health issues knows that doesn’t work. You need weekly sessions or something more consistent. Group 5 - Young people from the periphery

### 3.7 Change in future plans associated with climate change

Young people and women in peripheral areas significantly adjust their future plans as a result of the loss of material and economic assets due to the CC:

The amount of money spent to fix the roof [damaged by the storm]. Wow, almost 3,000 reais! The debt is still with the family to this day!!! I was going to leave the favela and study with that money, but that’s gone now. Group 1 - Young people from the periphery

In university students, it was observed that CC influences long-term plans, sometimes as a result of an intellectualized reflection on the global situation of the impact of CC.

Maybe it’s a bit extreme, but I’ve already decided that I don’t want to have kids. It’s a decision. It’s not the only factor, but it’s a very important one. I don’t want to bring a child into an environment like this. It’s a plan for the future, a view I’ve held for many years, and I don’t think I’m going to change. Group 6 - Young university students

### 3.8 Participation in individual and collective activities to combat climate change

All participants acknowledged the importance of combating CC. Although collective action was minimal, they were aware of and, as far as possible, practiced individual pro-environmental behaviors, such as recycling, water conservation, reducing electricity use, walking, using public transport and adopting a vegan diet (the latter limited to university students).

I have an Auntie Miriam… When it rains, she uses a technique, one that the people have developed… the rain falls on the roof tiles and then it falls into a bucket and she uses it to wash clothes. Sustainability. An old Black woman, you know? She’s not educated, she doesn’t have a degree. But she’s resourceful. Because I learnt about sustainability at my university, when I studied interior design. How do I make a sustainable project? If you have a lot of money, you can. But when I see Auntie Miriam doing it like that, I say: ‘Wow, it is possible!’ It’s just that we need to re-educate ourselves, you know. Group 3 - Young people from the periphery

Me too, I recycle, I separate the packaging, waste, plastics, I wash the packaging, everything is nice. I collect cans, not for myself, but I leave the cans there and put them in the rubbish bin so that someone else who needs them can pick them up, which I think is a great thing about recycling. And then I try to reuse the water. So, the water from the machine there I use to clean something, or to use in the bathroom. So I try to create these habits. Group 3 - Young people from the periphery

However, people from vulnerable communities faced challenges in adopting these behaviors due to their circumstances of poverty.

That’s it, because we need two things first, you can’t do anything if you’re hungry. So the first thing is to eat. The second thing is to have a home. And then, if you have the minimum structure, maybe I can think about the environment, the environmental issues of my planet. Group 5 - Young people from the periphery

Being sustainable, taking care of the world is very expensive. For example, regarding pollution, one solution to pollution is electric cars. But electric cars are very expensive. Healthy eating, without buying processed foods, is also expensive. A home in a safe area is too. And we, who are poor, can't do anything about it. Group 1 - Young people from the periphery

Young people and women from vulnerable communities criticized the environmental discourse of organizations that fail to reflect their realities. They believe that the focus should be on the industries and wealthier sectors responsible for pollution. These individuals feel pressured to adopt pro-environmental behaviors, but they have to prioritize daily survival amid extreme vulnerability, asserting that they lack the time to consider planetary survival:

There’s no way we can come to request or ask people to contribute to improving the environment, when we can’t even eat, our house falls down, our house floods, and demand these things, like, we don’t have the structure, we don’t have the knowledge. Greenpeace doesn’t come to the favelas to do grassroots work to talk about the climate changes that affect us, Greenpeace is talking about the businesses melting out there, it’s not about a panda, I’ve never seen a panda, it’s something else. Group 2 - Women community leaders

### 3.9 The role of the state, institutions and other agencies

All the groups emphasized the absence of support from institutions in addressing poverty and the impact of CC. Participants consistently highlighted a lack of government investment in environmental and climate education, believing that economic interests take priority over environmental preservation or restoration:

So I think that before thinking about climate change, everything we’ve talked about here is about the absence of the state, the absence of public policy, it’s about poverty. That’s it. What does the state do for the poor? The prefecture, the government, the municipality, the federal government? It doesn’t do anything, it’s very difficult, it’s very precarious. Group 2 - Women community leaders

It makes me very angry to see these people in positions of government, of more direct power, unable or unwilling to act on this. Group 4 - Young university students

Turn off the tap, take short showers. Why not tell the industry? Produce less, so we consume less, because everything that is produced is much more than we actually need. Why not tell shopping centers, 'Let's close on Sundays,' 'Let's close on Mondays.' Close one day a week! Not only will our work be less exploited, but it will also save a lot. Have you ever thought about what a shopping center with all those bathroom lights and water wastage is like? And then the guy looks at my face and tells me to take a five-minute shower. Have some respect, mate! These are absurd things! And it's all on our tab, right? It's the poor who pollute, the poor who throw rubbish in the street, the poor who live in risky areas, they're lazy, they don't like to work. But what do they do for the poor? Right?. Group 2 - Women community leaders

I think it’s a lack of encouragement, if someone doesn’t explain it to you, if someone doesn’t teach you, how are you going to learn that that’s where you have to throw it, that that’s how you dispose of something, or that if you take one thing, you can turn it into something else. These are the three most basic rules that are not practised in our daily lives: reuse, recycle and reduce. Because I know that the state, the government, has the resources to teach our population what’s right, to respect both us as human beings and nature itself, because that’s where our food comes from. Group 3 - Young people from the periphery

Watching the presidential debates, we could see that agribusiness was important to all the candidates. Nobody went head-on at them. I think agribusiness, if you look at any serious research on the climate crisis, is something that has an impact. And I don’t think there has been a government in the history of Brazil that has gone toe-to-toe with agribusiness. Group 4 - Young university students

The 4 years preceding this study were marked by Jair Bolsonaro’s presidency (2019–2022), whose environmental policies accelerated deforestation through deregulation and reduced enforcement, leading to record forest loss in the Amazon. Budget cuts to key agencies like IBAMA and ICMBio further weakened oversight. Bolsonaro’s government also promoted agribusiness and mining, facilitating the exploitation of protected and Indigenous lands while reducing fines for environmental violations, fostering impunity for illegal activities such as logging and forest burning. Additionally, his administration scaled back Brazil’s commitments to international agreements like the Paris Accords. These factors may have contributed to our participants’ heightened distrust off government institutions and their ability to address climate challenges effectively.

Further detailed information about additional complementary quotes of the participants is provided in Supplementary material, Annex F.

A synthesis of the key findings is provided in Supplementary material, Annex G.

## 4 Discussion

In this qualitative study, most participants demonstrated awareness of CC; however, a notable knowledge gap was identified between their understanding of the concept and its underlying mechanisms. Our results are in line with other studies (23–25). Most participants from vulnerable communities defined CC as an increase in local temperatures and unpredictable shifts in weather patterns, but also included in the definition its consequences, such as floods, storms, landslides, hurricanes, wildfires and the proliferation of insects and rodents in new locations. Including extreme climate events in the definition of CC is understandable, as individuals’ understanding of CC, especially in vulnerable communities, is often associated with direct lived experiences rather than with formal education. Conversely, participants who were university students from middle- to high-income backgrounds primarily acquired their CC perception through the classroom and media sources (internet, television programs, etc.). Our study also revealed varying levels of knowledge about CC, with some participants from poorer areas admitting that they did not understand its causes. A qualitative study of 107 Indonesian youth (aged 15–29) also found that, while they recognized the climate crisis, their understanding of its driving mechanisms was limited (23).

Although participants in our study were unfamiliar with the term “eco-anxiety,” they clearly expressed sentiments consistent with its definition. Hence, participants in socioeconomically disadvantaged areas exhibited the highest emotional responses—constant concern, fear, anger and hopelessness—due to frequent and severe climate crises that directly impact their lives. Conversely, university groups showed less intense emotional reactions—such as anxiety, helplessness and resignation in the face of distant CC events. They also reported feeling powerless, which widens the gap between knowledge and action, potentially intensifying feelings of hopelessness (26). Recent studies corroborate these findings. A survey conducted with 10,000 young people (aged 16–25 years) from 10 different countries revealed that 59% were extremely worried about CC and 75% viewed the future as frightening (6). Several studies have indicated that personal experiences of CC are associated with perceptions of risk (27) and heightened CA (7, 12). The connection between CA and CC is quite complex, as highlighted by diverse findings across 32 countries (13).

An essential observation derived from our study indicates a clear correlation between socioeconomic inequalities and the risk of suffering an adverse CC event. Participants from vulnerable communities describe traumatic, sometimes life-threatening, experiences resulting from the direct impact of CC events. A clear perception of environmental injustice was universally recognized across all participant groups in our study. Participants emphasized that those living in poverty experience the adverse consequences of CC to a disproportionate degree. Conclusions drawn from a systematic review (3) highlighted that younger generations, women, and rural and Indigenous populations from economically disadvantaged nations are more susceptible to extreme CC events. Similarly, a 2023 study carried out in 10 countries from the Global North and the Global South (Australia, Brazil, Finland, France, India, Nigeria, Philippines, Portugal, the UK and the USA) found that respondents in the Philippines, India and Nigeria reported a stronger psychological impact of CC than respondents in the USA and Finland (10). An integrative review asking “how does Generation Z experience ecological and climate crisis” Tsevreni et al. (28) came to the similar conclusion that Generation Z worries in the Global North but suffers in the Global South.

Gender-based vulnerability (GBV) increases susceptibility to CC (29). This study found that Black women leaders view CC as worsening existing social inequities, including racial and gender-based violence. This aligns with previous research that indicates that extreme events increase GBV due to factors like economic instability and food insecurity (29). An Indian survey revealed that women in drought-affected areas face a higher likelihood of partner violence than those in non-drought areas (30). Moreover, the Intergovernmental Panel on CC Sixth Assessment Report (31) acknowledges that CC disproportionately affects marginalized racial and ethnic groups, exacerbating existing social inequalities. This finding underscores the importance of adopting both gender- and race-sensitive approaches to effectively manage the impacts of CC.

In our study, participants from poorer areas experienced the most pronounced impact of CC on both physical and mental health, reporting higher incidences of respiratory and infectious diseases, panic attacks and PTSD. University students, on the other hand, linked awareness of distant CC events to anxiety and depression, with some also mentioning the need for therapy. A scoping review of 10 reviews carried out in the Western Pacific Region showed increased PTSD and decreased mental well-being in response to droughts, floods, storms and climate migration (32). In New Zealand, the adult population linked CC concerns to poorer psychological well-being over a year (33), and 12,246 university students from 32 countries identified a significant association between CA and mental well-being (13). Furthermore, individuals with pre-existing mental health diagnoses were at higher risk of psychopathological symptoms and a depressive response to CC (34).

In more precarious peripheral areas, familial and community networks are vital for resilience against climate crises, providing emotional support and resources for adaptation and reconstruction. However, vulnerable populations struggle to access long-term mental health treatments through the SUS, underscoring the need to improve mental health services in response to extreme climate events. Access to healthcare remains inadequate in Brazil, especially in remote and impoverished regions, despite progress in addressing mental health issues (35). Our findings are consistent with previous research on family and psychotherapy support. A qualitative study in Quebec, Canada, found that family and friends are crucial for coping with CC anxiety, helping young people feel less helpless and promoting proactive attitudes toward CC (36). Psychotherapy also provides support against the impacts of CC. A study in Sweden found that discussing CC-related distress in therapy helps individuals manage their emotions, though therapists should have some knowledge of CC (37). Another scoping review found that focused work groups can provide emotional support for those with CC anxiety (38).

Our study found that CC has abruptly disrupted the future plans of some vulnerable populations due to family or material losses and health impacts. These findings align with other studies. For example, vulnerable women in sub-Saharan Africa reported that changes in precipitation and temperature reduced their desire for children (39) CC has also been linked to impacts on agricultural production, fertility rates, fetal development, and obstetric outcomes in affected regions (40, 41). In our research, university students’ future decisions, including the choice not to have children, were influenced by more long-term concerns related to CC. Similarly, studies in Sweden and Switzerland have found that CA is associated with negative attitudes toward childbearing (42).

While all participants in this study recognize the urgent need to address CC, there is a notable lack of engagement in collective pro-environmental actions. The vulnerable population expressed a dual sense of environmental injustice. They argue that pro-environmental messaging overlooks their increased vulnerability and fails to demand action from major contributors to CC. Additionally, they feel marginalized, as environmental discourse does not provide training or strengthen their ability to respond to frequent environmental crises, rather seeming disconnected from their lived experiences. Moreover, CA could drive pro-environmental action, but this again is limited by lack of resources, knowledge and opportunities (43).

Therefore, integrating environmental initiatives with programs that address basic needs, such as food security, safe homes, healthcare and employment, alongside educational programs on CC, can be more effective. Thus, in wealthier countries, individuals more often engage in individual pro-environmental actions based more on knowledge, opportunities and personal convictions than in collective actions (13, 44). These behaviors, typically learnt at home from family, provide satisfaction (36). However, young people often see them as ineffective due to low self-efficacy (24, 45) and believe that collective action on CC should be led by governments, emphasizing the need for public education, law enforcement, policy development and investment in research and technology (24).

Participants in this study unanimously highlighted the lack of intervention by public authorities in addressing CC. They emphasized the absence of government investment in environmental education, infrastructure improvements and policies to reduce social inequalities, which are essential for mitigating the severe impacts of CC. Participants felt that economic interests were prioritized over meaningful environmental actions, leaving them feeling abandoned and unsupported by authorities. Similarly, studies of young people have confirmed the existence of feelings of distress and betrayal due to governmental inaction (6, 46).

Finally, the nine themes identified in this study align with Sustainable Development Goals (SDGs) 3, 10, 11, and 13, emphasizing the interconnectedness of CC, health, inequality, and urban sustainability. Regarding health and well-being (SDG 3), eco-anxiety and other climate-related emotions affect mental health, while extreme weather events exacerbate physical conditions such as respiratory, infectious, and heat-related illnesses, particularly in vulnerable communities with limited access to healthcare. In terms of reducing inequalities (SDG 10), CC deepens social disparities, disproportionately affecting marginalized populations with fewer resources to cope with environmental crises. Concerning sustainable cities and communities (SDG 11), participants’ experiences highlight urban challenges in underserved areas, including poor sanitation and unsafe housing, while also underscoring the role of support networks and civic engagement in fostering resilience. In relation to climate action (SDG 13), perceptions of inadequate institutional support in addressing poverty and CC impacts, shifts in future planning, and limited resources to engage in collective climate initiatives were identified as critical barriers to effective mitigation and adaptation strategies. These findings underscore the urgency of integrated policies that simultaneously address the climate crisis and its social repercussions.

### 4.1 Limitations and proposals for future studies

While this study provides valuable insights into eco-anxiety, inequality, and environmental injustice in São Paulo, certain limitations must be acknowledged. First, as a qualitative study based on focus groups, the findings are not meant to be generalized beyond the specific populations studied. The perspectives shared by participants provide rich contextual understanding but may not fully capture the diversity of experiences within other vulnerable or privileged communities in Brazil.

Second, due to word count limitations, we could not include the discourse of all groups in each thematic section of the results. However, each section presents a summarized common response that represents the general view of all groups. While some nuances may not be fully detailed, we ensured that the essential perspectives and key themes were preserved. We do not believe that this approach resulted in the omission of distinct or critical information.

Third, the sample primarily included young people and Black women leaders, which, while essential to the study’s aims, does not account for other vulnerable groups, such as older adult populations, people with disabilities, who may have distinct experiences of CC impacts.

Fourth, the study relies on self-reported experiences and emotions, which may be influenced by recall bias or social desirability bias, particularly when discussing personal coping mechanisms and mental health challenges.

Future studies should adopt a participatory, co-creation approach to develop and implement interventions aimed at enhancing resilience and coping strategies in vulnerable communities. Collaborative research involving community members, local organizations, and policymakers can help design context-specific programs that address both the psychological and structural challenges of CC. This could include mental health interventions tailored to eco-anxiety, environmental education programs, and community-driven adaptation strategies.

Additionally, longitudinal research is needed to assess how climate anxiety and resilience evolve over time, particularly as climate-related events intensify. Quantitative approaches could complement qualitative data by systematically measuring the psychological and health impacts of CC.

By integrating co-creation methodologies and intervention studies, future research can contribute to the development of sustainable community-led solutions that empower individuals and strengthen collective resilience against climate-related challenges.

### 4.2 Summary of the discussion

This study found that poverty and social inequality are central to understanding population vulnerability and limited adaptive capacity to CC, echoing the vulnerability factors outlined in the conceptual framework. Participants described clear cases of environmental injustice, with low-income communities facing disproportionate exposure to climate crises and lacking access to adaptive resources—key conditions that heighten eco-anxiety and mental distress.

Individuals in socioeconomically disadvantaged areas reported more intense emotional responses and more severe physical and mental health impacts compared to university students. These effects were often tied to direct exposure to climate events—such as damage to housing or health emergencies—supporting the framework’s focus on direct stressors as drivers of eco-anxiety. In contrast, university students experienced eco-anxiety primarily in response to indirect or perceived threats, encountered through media and academic discourse. This aligns with the conceptualization of anticipatory worry and cognitive rumination as key components of eco-anxiety in populations less directly affected.

Social determinants—such as race, gender, and geography—emerged as critical, intersecting factors shaping vulnerability. Women in disadvantaged areas, in particular, reported compounded risks during climate events due to experiences of racial and gender-based violence. These findings reinforce the framework’s identification of marginalized populations as especially susceptible to the health impacts of CC, particularly those affecting mental well-being.

Familial and community networks served as vital informal coping mechanisms, particularly among vulnerable populations, highlighting the importance of social support as a key buffer against eco-anxiety. However, limited access to long-term mental health care within the SUS reveals persistent health inequalities and exposes systemic gaps in the formal resilience infrastructure. These shortcomings underscore the urgent need to strengthen institutional supports capable of effectively mitigating the psychological impacts of CC.

The findings also reflect the functional outcomes of eco-anxiety. In disadvantaged communities, material and health impacts led to disruptions in education and employment plans—illustrating life function impairments and restricted future planning. In contrast, students reported lifestyle shifts such as delaying parenthood, suggesting how perceived threats and cognitive stressors influence long-term decision-making, as noted in the framework’s outcome domain.

Despite a shared recognition of the urgency of climate action, engagement in collective pro-environmental behaviors remained limited—particularly among vulnerable populations, where immediate economic survival often took precedence. These constraints reflect the vulnerability factors identified in the conceptual framework, such as poverty, limited access to information and resources, and broader structural inequalities that restrict adaptive capacity. The findings underscore how systemic barriers—rooted in socioeconomic disadvantage—impede both climate adaptation and participation in sustainability efforts, further exacerbating climate-related vulnerability.

## 5 Conclusion

In conclusion, these findings highlight the critical need for inclusive knowledge co-production policies—approaches that deepen understanding of local realities, build trust, and strengthen community agency, particularly in marginalized contexts. More broadly, the study underscores how systemic social and health inequalities intersect with the psychological, emotional, and behavioral dimensions of eco-anxiety, shaping individuals’ lived experiences of CC. To build genuine resilience, public policies must be locally grounded, socially inclusive, focused on poverty reduction, and rooted in principles of environmental justice. Addressing these structural challenges is not only a matter of equity but also a public health imperative—essential for promoting mental well-being and enabling sustainable adaptation in the face of CC.

## Data Availability

The original contributions presented in the study are included in the article/Supplementary material, further inquiries can be directed to the corresponding authors.
