# Supplementary material for: “No forest, no future, but they don’t see us”: eco-anxiety, inequality, and environmental injustice in São Paulo
Source: Front Public Health. 2025 Jun 5;13:1555386. doi: 10.3389/fpubh.2025.1555386 (PMC12176893; doi:10.3389/fpubh.2025.1555386)
Supplement: Supplementary file 1 [file Data_Sheet_1.docx]

**Annex A. Eco-Anxiety: A Conceptual Framework from a Public Health Perspective**

**1. Eco-Anxiety: A Multi-Dimensional Construct**

Eco-anxiety is understood as a **complex psychological response** to climate change, encompassing:

- **Emotional distress**:
  - Feelings of fear, sadness, guilt, helplessness
- **Cognitive processes**:
  - Rumination, anticipatory worry, catastrophic thinking
- **Somatic symptoms**:
  - Physical tension, fatigue, somatic complaints
- **Functional impairment**:
  - Sleep disturbances, reduced concentration, impact on daily functioning

**2. Stressors / Triggers**

Eco-anxiety arises from both direct and perceived climate change threats:

- **Direct climate change (CC) events**:
  - E.g., wildfires, floods, heatwaves, droughts
- **Perceived threat of future events**:
  - Shaped by:
    - **Media exposure**
    - **Educational content**
    - **Lived experience or family history**

**3. Vulnerability Factors**

Certain populations are more susceptible to eco-anxiety due to **social determinants of health**:

- **Socioeconomic disadvantage**:
  - Limited access to healthcare, information, or adaptive resources
  - Greater exposure to environmental hazards
- **Marginalized or racialized populations**:
  - Black, Indigenous, and other racialized communities
- **Environmentally dependent communities**
- **Youth**:
  - Especially those in adolescence or early adulthood
- **Gender**:
  - Higher prevalence reported among women
- **Geographical vulnerability**:
  - Populations in the **Global South**, disproportionately affected by CC

**4. Moderators / Mediators**

These factors can **amplify or buffer** the effects of eco-anxiety:

- **Pro-environmental behavior and climate activism**:
  - Can foster a sense of agency and reduce helplessness
- **Belief systems**:
  - E.g., “green self-identity,” ecological values
- **Perceived efficacy**:
  - Belief in personal or collective (e.g., government/NGO) capacity to make a difference
- **Social support**:
  - Family, community, peer support systems
- **Access to mental health care**

**5. Outcomes (Public Health Impacts)**

**Mental health outcomes**:

- Anxiety, depression, insomnia, PTSD-like symptoms

**Functional outcomes**:

- Cognitive impairments (e.g., poor concentration)
- Daily life disruptions (e.g., work, study, social functioning)

**Behavioral responses**:

- **Adaptive**: increased climate engagement, activism, lifestyle changes
- **Maladaptive**: paralysis, avoidance, emotional numbing

**Lifestyle decisions**:

- Reproductive choices, relocation decisions, life satisfaction, future planning
