# Supplementary material for: “No forest, no future, but they don’t see us”: eco-anxiety, inequality, and environmental injustice in São Paulo
Source: Front Public Health. 2025 Jun 5;13:1555386. doi: 10.3389/fpubh.2025.1555386 (PMC12176893; doi:10.3389/fpubh.2025.1555386)
Supplement: Supplementary file 2 [file Data_Sheet_2.docx]

**Annex B. Script for Focus Groups**

All participants received an allowance of R$100 for any expenses related to taking part in the study, and the community leaders responsible for recruiting the participants were reimbursed R$200.

**Presentation and warm-up (10 minutes)**

**General presentation:** The interviewers introduced themselves, outlining their professional backgrounds and the main goal of the research. They provided information on data protection and research ethics, emphasizing confidentiality and academic purpose. Participants were reassured that there are no right or wrong opinions and that all perspectives are welcome and encouraged throughout the discussion.

**Warm-up**: round of introductions of participants.

**Part 1 (20-25 minutes) Perceptions, Feelings and Experiences of Crises/Climate Change**

*In this* **1st** *part,* *we will explore:*

Perceptions (knowledge) about climate change and what phenomena they associate with climate change.

Feelings and emotions about climate change.

Lived experiences of the direct impact of acute meteorological phenomena/disasters and how they have affected life.

**Questions and prompts:**

Today we’re going to discuss climate change and its impact on our lives:

What do you think of when I say, ‘climate change’?

[discussion round]

**Visualization of selected images (20-25 minutes)**

We offer 10 images related to the theme. We ask you to choose the image that best represents climate change.

**Questions and prompts:**

- I will then ask you to select from the images in these individual envelopes some that depict problems related to climate change. Please do the work individually as we want to get to know the different points of view. We would remind you that there is no consensus on this issue, no right or wrong, what we are interested in is your opinion.

o *Explore the feelings and emotions triggered*

o *Explore negative feelings (anxiety, fear, anger, powerlessness and other colloquial terms, etc.) and positive feelings.*

- Choose one that you perceive as more expressive and tell us why?

- Why do you think it happens?

What do you feel when you see these images about climate change?

o *Explore concrete experiences and encourage discussion about the severity of the events, how much it has affected your life and the community where you live, and whether the frequency has increased in recent years*.

- For some people climate change is a subject that doesn’t affect them directly, for others it’s something that has already affected their lives or may do so. Has anyone here experienced a situation that they think is related to the effects of climate change? Please describe it.

**2nd part (25-30 minutes). Knowledge about the concept of eco-anxiety, impact on health, support from networks and institutions, change and future plans**

Now we talk about what you know about the term eco-anxiety and who among you has experienced it, in what way and what your experiences have been:

o *Explore impact of CC on general health and mental health.*

*o Explore frequency and impact on daily life.*

**Questions and prompts:**

- What do you think when I say ‘eco-anxiety’?

- You think that climate change could affect your health or well-being. In what way?

-For those of you who haven’t experienced such situations directly, how do you feel when you hear that other people have been affected by climate change? Does it affect you in any way? How does it affect you?

o *Explore health impacts, including depression, anxiety, post-traumatic stress disorder, etc. and what kind of support they have received.*

**Questions and prompts:**

- Did it affect your health?

-And your mental health, how?

-Did you have any support? Which kind?

o *Explore personal, family, and local community support networks*.

Explore (national and international) institutional and government support.

**Questions and prompts:**

- How did you manage situations arising from the impacts of climate change?

-Did you have support from family, friends, neighbours…?

**-** Do you think the government (local, national) and international organizations are doing anything to reduce climate change? What ?

**Questions and prompts:**

- Have you changed any of your plans for the future as a result of your experiences/feelings of climate change?

**Part 3 (20-25 minutes) Social inequalities, vulnerability, resilience, pro-environmental behaviours**

In this final section we will explore the dimensions of social and global awareness of the problem, as well as learning about the population’s capacity for adaptation and resilience to the climate crisis.

**Climate change: vulnerabilities, resilience and coping strategies**

o *Explore proactive attitudes to protecting the environment and whether they have any relevance to reducing individual eco-anxiety.*

**Questions and prompts:**

- Do you think everyone suffers equally from climate change in the world?

- And here in São Paulo, do you think everyone suffers equally?

- Have you been involved in individual and/or collective activities to protect the environment?

- How do you feel about being part of these activities?

**Final block**

Would anyone like to add anything else?
