# Supplementary material for: “No forest, no future, but they don’t see us”: eco-anxiety, inequality, and environmental injustice in São Paulo
Source: Front Public Health. 2025 Jun 5;13:1555386. doi: 10.3389/fpubh.2025.1555386 (PMC12176893; doi:10.3389/fpubh.2025.1555386)
Supplement: Supplementary file 3 [file Data_Sheet_3.docx]

**Annex C**: Socio-demographic and health form.

**How old are you? ______________________________________**

**How do you identify with your gender?**

- Cis-gender man
- Cis-gender woman
- Transgender man
- Transgender woman
- Non-binary person
- Other _________________________________________

**How do you identify with your sexual orientation?**

- Homosexual/gay
- Lesbian
- Heterosexual
- Bisexual
- Pansexual
- Other ________________________________________

**How do you identify with your race/skin color?**

- White
- Black
- Brown
- Indigenous
- Yellow
- Other _______________________________________

**Are you currently studying?**

- Yes
- No

**Until which grade did you go to school?**

- I didn’t go to school
- 1st to 3rd grade / currently 1st to 4th grade
- 4th grade / completed primary school / currently 5th grade
- 5th to 7th grade / incomplete gymnasium / currently 6th to 8th grade
- 8th grade / completed elementary school / currently 9th grade
- 1st or 2nd grade of high school / high school incomplete
- 3rd grade / high school completed
- Higher education incomplete
- Complete university degree
- Post-graduate degree (complete/incomplete)
- NS/NR

**How many people live in your house (including you)? _________________________________**

**Who is responsible for the maintenance of your home? _______________________________**

**Do you work? If so, what do you do? ______________________________________________**

**Do you currently have any illnesses?**

- No
- Yes, which one? ______________________________________________

**Do you take any medication regularly?**

- No
- Yes, which one? ____________________________________________

**Have you been diagnosed by a health professional with any of these conditions? Please indicate all that apply.**

- Depression
- Panic attack
- Anxiety
- Post-traumatic stress disorder
- Other? _________________________________________________
- None of these conditions

**Do you take medication for any of these conditions?**

- Yes
- No

**In the last month, have you felt sad or depressed most days?**

- Yes
- No

**Why do you think you feel this way? _________________________________________________________________________________________________________________________________________________________________________________________________________________________________**
