# Supplementary material for: “No forest, no future, but they don’t see us”: eco-anxiety, inequality, and environmental injustice in São Paulo
Source: Front Public Health. 2025 Jun 5;13:1555386. doi: 10.3389/fpubh.2025.1555386 (PMC12176893; doi:10.3389/fpubh.2025.1555386)

**Annex D.**  Trigger images used in the focus group sessions.

1. Flooding and homelessness


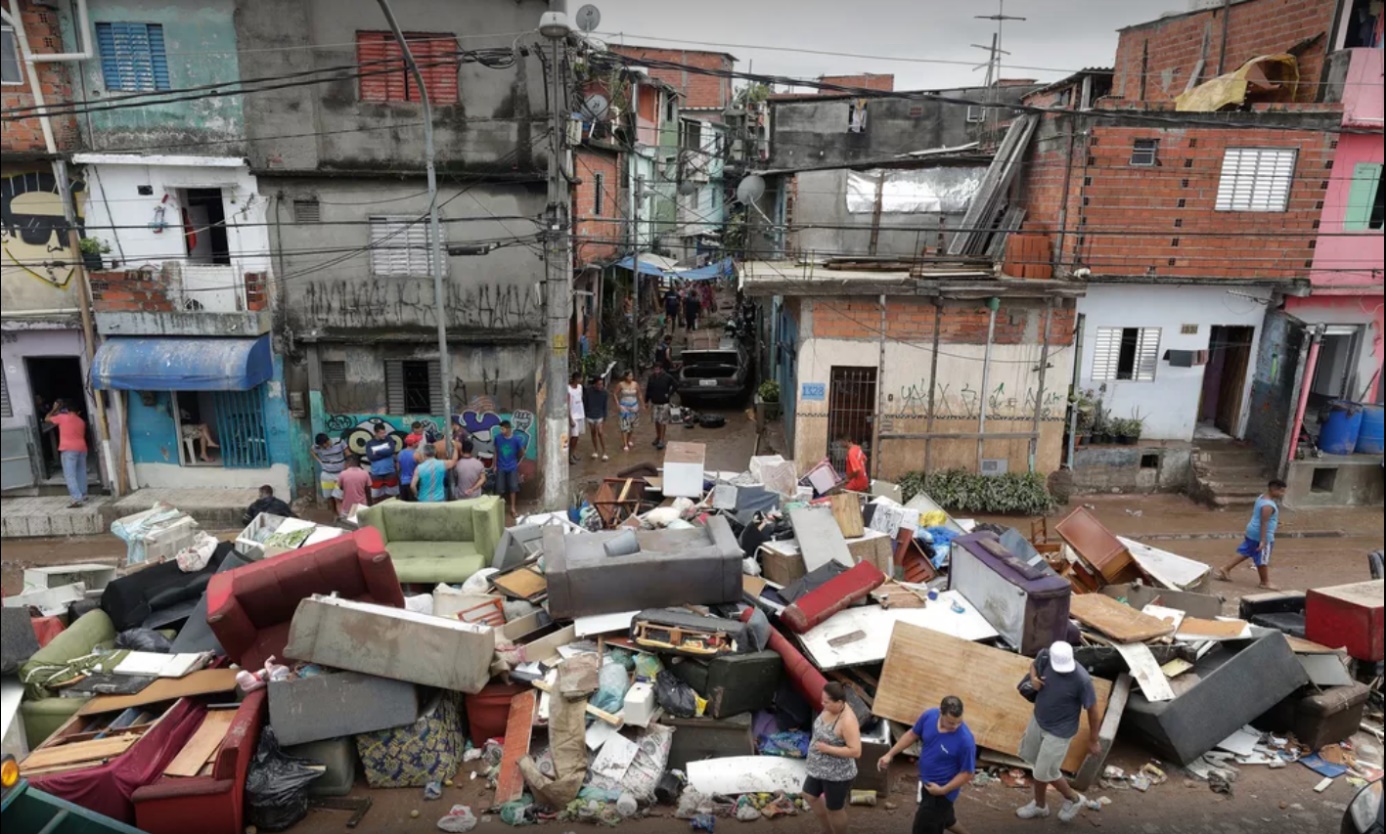


1. Landslides


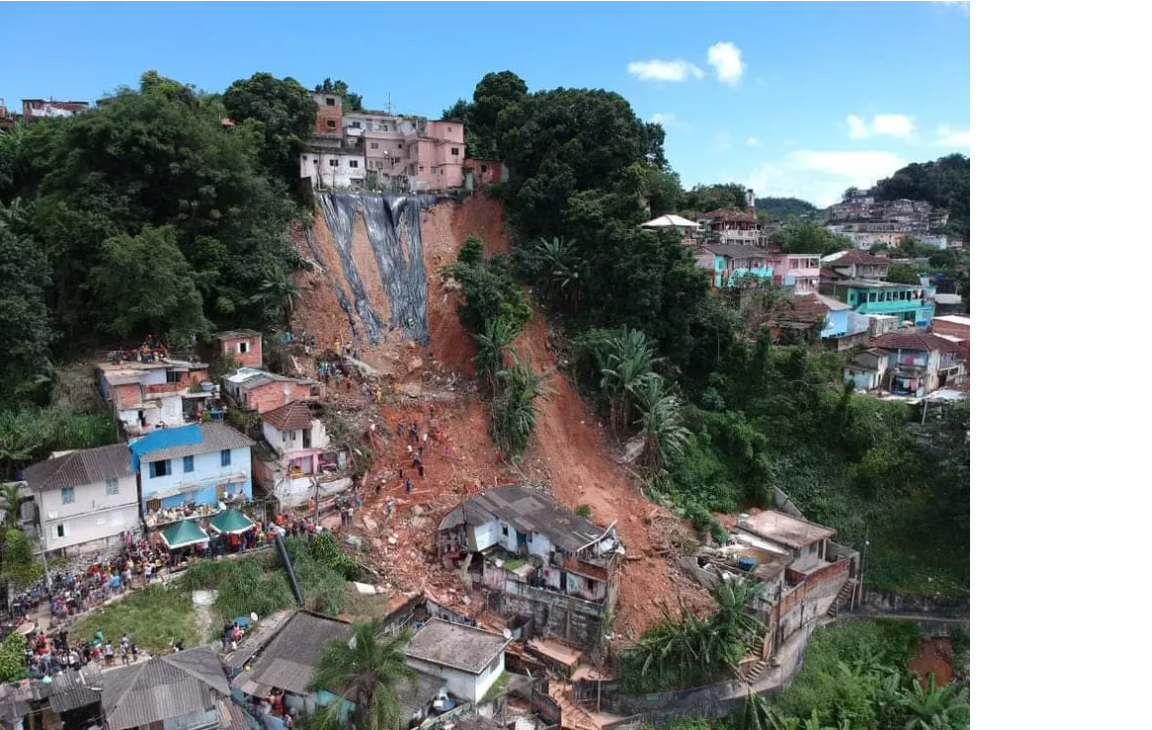


1. Polar melting


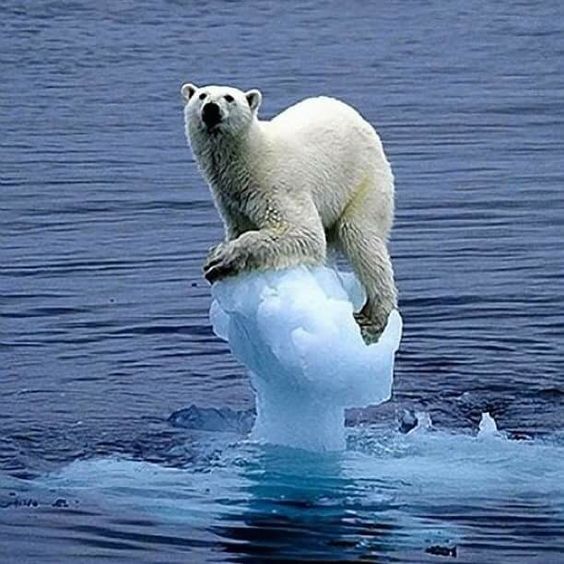


1. Concerns about climate change


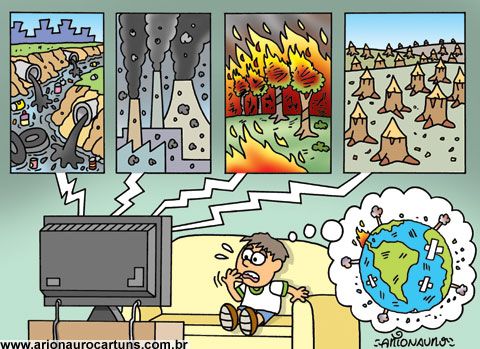


1. Protests for the future of the planet


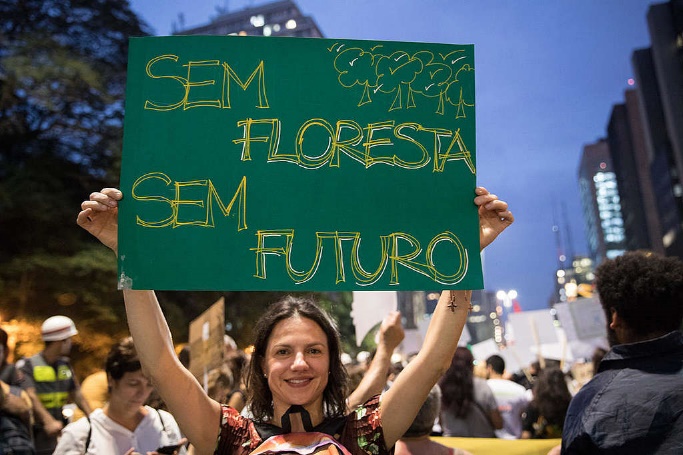


1. Floods and migration


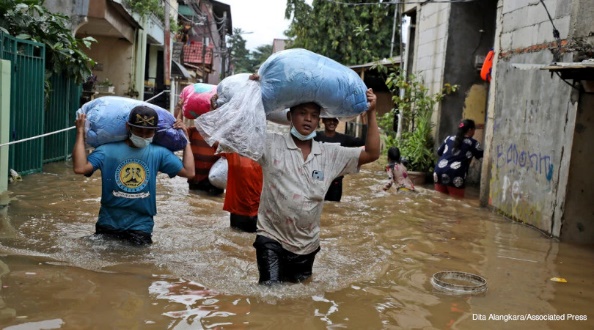


1. Floods and loss of infrastructure


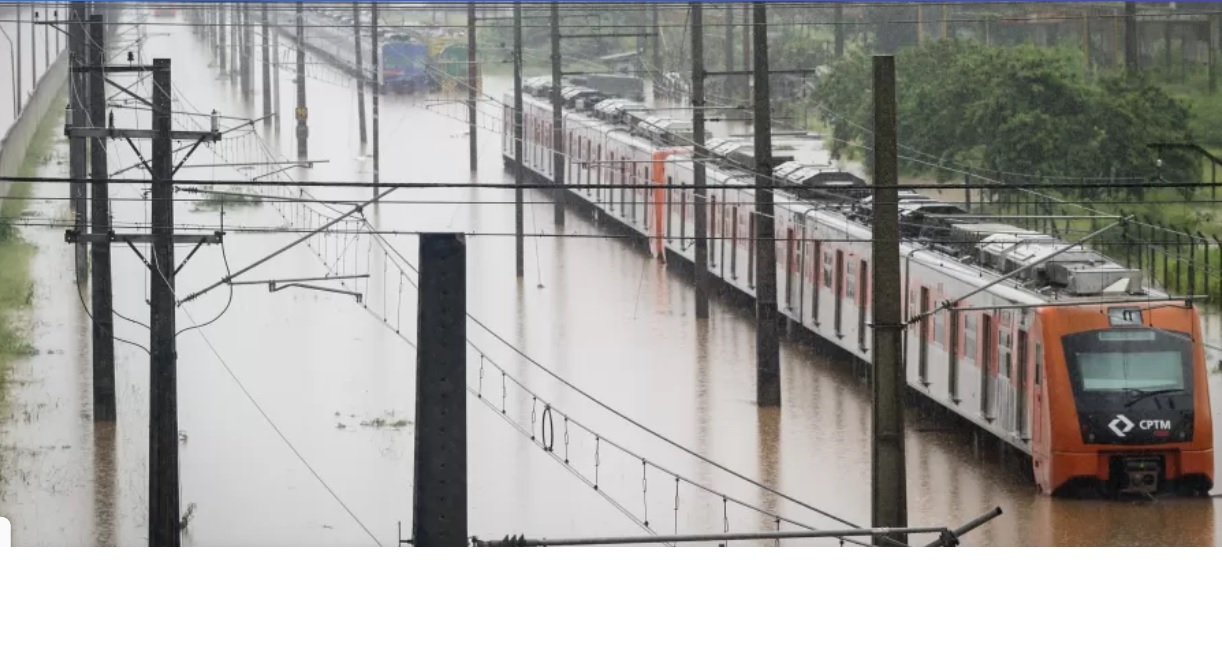


1. Wildfires


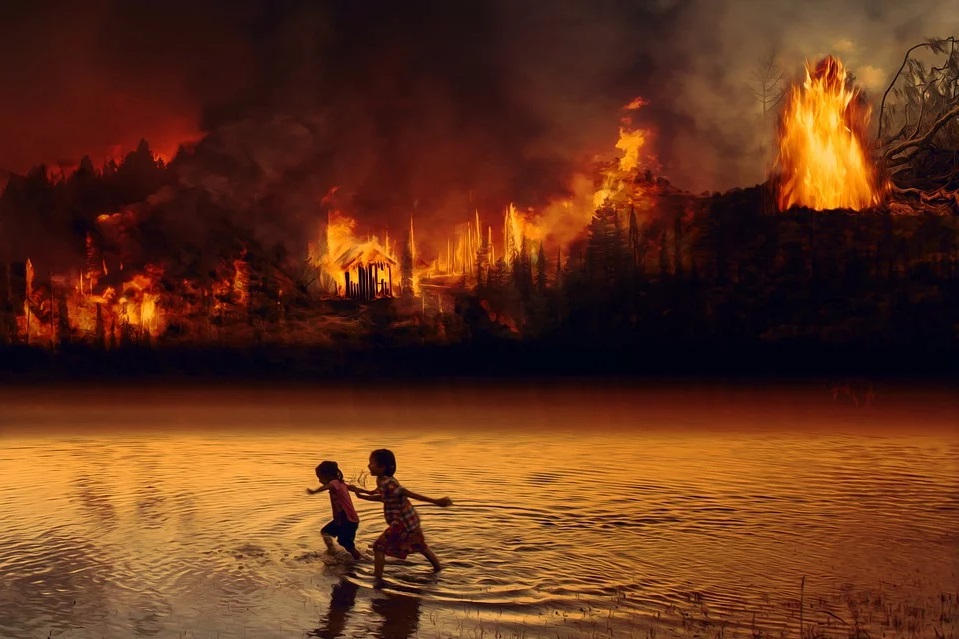


1. Polluting rubbish


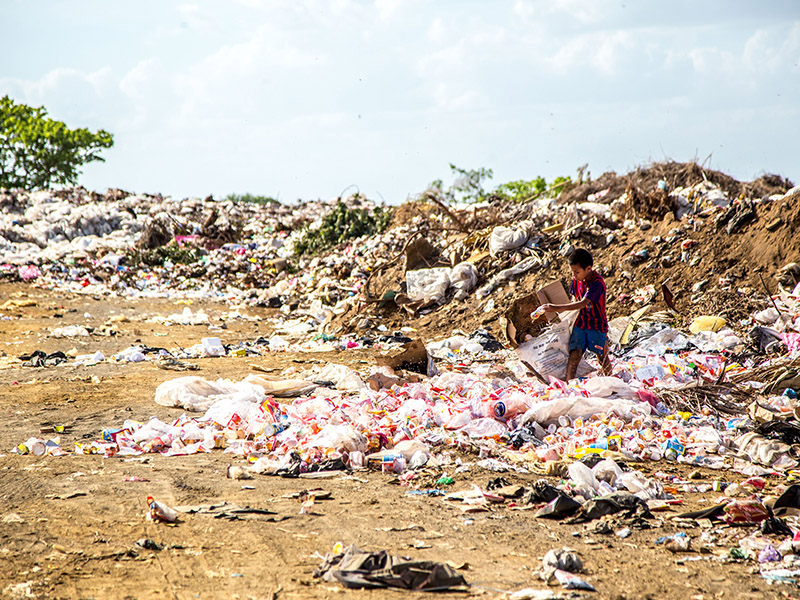


1. Sustainability of the planet


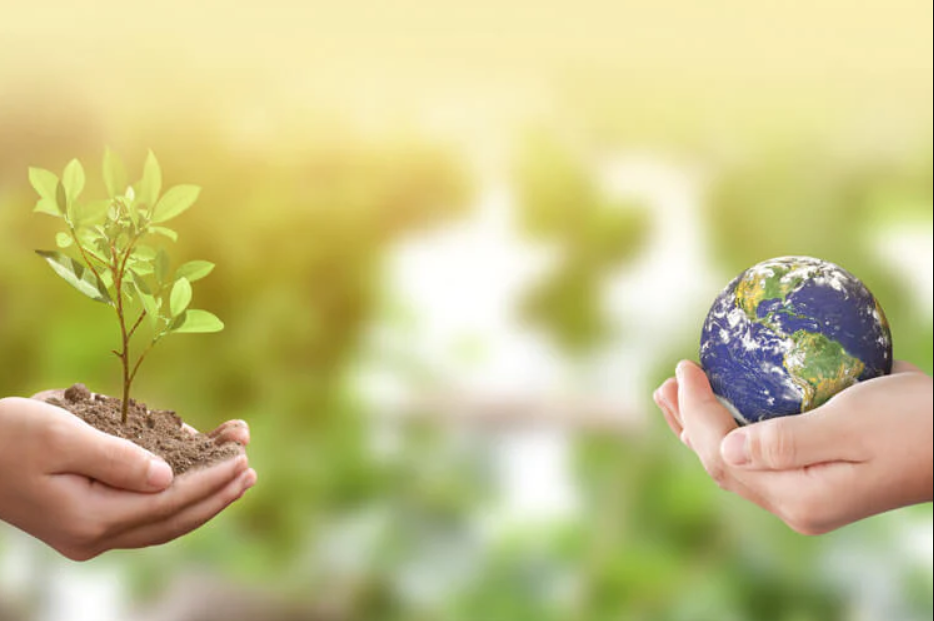

Supplement: Supplementary file 4 [file Data_Sheet_4.docx]
