# Supplementary material for: “No forest, no future, but they don’t see us”: eco-anxiety, inequality, and environmental injustice in São Paulo
Source: Front Public Health. 2025 Jun 5;13:1555386. doi: 10.3389/fpubh.2025.1555386 (PMC12176893; doi:10.3389/fpubh.2025.1555386)
Supplement: Supplementary file 5 [file Data_Sheet_5.docx]

**Annex E. Sociodemographic and health characteristics of the study participants**

In total, 62 people participated in the 6 focused groups. Each focused group consisted of 9 to 12 participants: 3 groups of suburban youth (Groups 1, 3, 5; n=29), 2 groups of university students (Groups 4, 6; n=22) and 1 group of Black women community leaders (Group 2; n=11). The mean age of all participants was 23.5 with an interval of 18 to 59 years.

The majority (38/62) identified as cisgender women, followed by 22/62 as cisgender men. In turn, of the 62 people, 36 were heterosexual and 7 were bisexual. Participants were mostly dark-skinned (30 people, 48%), followed by White (24, 39%), Asian (4, 7%) and Indigenous populations of the Americas (2, 3%) except for 2 participants who did not identify with any race (3%). The majority, 28 (45%) had incomplete tertiary education (less than 12 years) followed by 22 (35%) who had secondary education, 9 (15%) had tertiary education (undergraduate and graduate), and 3 (5%) had primary or lower education (2 of them with a duration of 5 years and 1 of them from 6 to 8 years). In addition, 24 (39%) had some job or occupation at the time of the study.

In relation to health status, 12 participants (19%) reported suffering from a chronic disease, among which the following stood out: allergic rhinitis (5%), migraine (3%), and hypertension (3%). In addition, of the 12, 3 people reported suffering from 2 or more of these conditions and 8 of the 12 participants took medication on a regular basis.

In relation to mental health, 38 (61%) reported having a clinical diagnosis of mental disorder, although only 8 (13%) were following systemic therapy. 15 (24%) reported a single diagnosis (12 reported anxiety, 2 depression, and 1 had post-traumatic stress disorder) and 9 (15%) reported a combination of more than one diagnosis (1 panic attack and anxiety, 1 panic attack and acute stress disorder, 1 anxiety and bulimia, 1 anxiety and bulimia; 1 depression and Obsessive Compulsive Disorder (OCD); 1 panic crisis, anxiety and post-traumatic stress disorder (PTSD); 1 depression, anxiety and panic crisis; 1 depression, anxiety and attention deficit hyperactivity disorder (ADHD); 2 depression, anxiety and borderline personality disorder). Finally, 28 participants (45%) reported feeling sad or depressed most days during the last month.
