# Supplementary material for: “No forest, no future, but they don’t see us”: eco-anxiety, inequality, and environmental injustice in São Paulo
Source: Front Public Health. 2025 Jun 5;13:1555386. doi: 10.3389/fpubh.2025.1555386 (PMC12176893; doi:10.3389/fpubh.2025.1555386)
Supplement: Supplementary file 7 [file Data_Sheet_7.docx]

**Annex F.** **Additional complementary quotes**

**3.1 Perceptions (knowledge, belief) about CC**

“.....*affects the amount of rain we have, it’s not normal*...” – Group 4 – Young university students

“... *I have the sense that when people talk about climate change, it’s all about catastrophes: melting glaciers, natural disasters, tornadoes, hurricanes, floods. It’s a sort of catastrophic perception that I have*.” - Group 6 - Young university students

## 3.2 Feelings and emotions related to CC

“*I get really anxious. It rains when it shouldn’t, and I need to leave here an hour earlier to get to the University Hospital, but everything’s at a standstill! It makes me super anxious*”. - Group 6 - Young university students

“*I’m eighteen and I feel fear, sadness, and anger, helplessness; if it keeps going like this, there won’t be a world left for us to live*”. - Group 3 - Young people from the periphery

“*I’ll be brief. What I feel when I think about the flooding, about these tragedies, is anger, a feeling of unfairness, of misery, of a lack of structure. Seeing people dying. Why is it happening? Why does all this happen and how can we stop it from happening, you know? So at the moment my thoughts are of anger, injustice and enormous sadness.”* – Group 1 - Young people from the periphery

“*I started to think about the environment, just in relation to teaching, when I started to see it at school and thought: ‘Wow, this exists here!’; ‘Wow, this happened here on such and such a day, that rain could be linked to it’. And that gave me a lot of anxiety, but at the same time, as I learnt more about it, I think it gave me a sense of hopelessness.”* - Group 6 - Young university students

## 3.3 Concrete experiences and perceptions associated with CC

*“Bro, the gully floods so fast*”. - Group 5 - Young people from the periphery

“*Wow, very sad! I wanted to cry. She fought so hard to build that house. She suffered a lot and she was heartbroken. She sent a message, she was totally beside herself because she lost her sofa, she lost her bed, she lost her son’s school materials, she lost her son’s clothes. She wasn’t at home, she was working. She didn’t have any way of helping, pushing, finding out who’s responsible.*”– Group 2 - Women community leaders

*...I don’t remember how old I was, but I was a bit younger, I must have been around 12, 13 years old. There was a rainstorm, there had never been rain or wind like that. Then I remember I was here in the kitchen, and there’s a window that looks out in front of my house, where the yard is. Then I saw the first wall fall. Then the second wall fell. Then I thought: ‘I'm going to die’. At that moment I started to get really scared, I thought: ‘My God, this house is going to collapse with people inside...’. Then I... the next day, we went to talk to other people, people who whose houses really did collapse. People had been swept away, cars had been swept away, trees had come down, people had lost everything. And then I thought: ‘I had it a bit less bad because only the wall fell...”.* - Group 5 - Young people from the periphery

## 3.4. Perception of social inequalities and their relationship with CC

“*The whole world isn’t affected in the same way, the lower classes are harder hit because living sustainably, looking after the world, is very expensive. For example, on the issue of pollution, it’s... one solution to pollution is electric cars, electric vehicles. But an electric car is a lot of car. Healthy eating, without buying industrial food, is also expensive. A safe place to live is also expensive. And those of us who are poor can’t do anything about it. And the rich, most of them at least, they don’t care, you know?*” - Group 3 - Young people from the periphery

“*Climate change is happening all over the planet, but people are feeling it differently depending on their status in society*.” – Group 5 - Young people from the periphery

“*It’s always the poor who suffer because we’re always in the wrong place. Why’s that? We’re in the wrong place because there’s nowhere to put a house, nowhere to build*.” – Group 1 - Young people from the periphery

## 3.5 CC and Impact on health

“*Wading through... floodwater, at risk of catching Weil’s disease*”- Group 2 - Women community leaders

“... *I go to therapy, which has helped me a lot, because I used to suffer a lot, I remember, I used to have anxiety attacks, when I thought (inaudible), at school, and I’d see these pictures in the geography textbook, I’d start having anxiety attacks, almost a panic attack, and I’d feel really unwell...”* - Group 6 - Young university students

## 3.6 Networks support to face severe climate crises

“.. there are 7 of us siblings, so when it rained and water came into the house, one would grab a bucket and the other would throw the water outside... because there was so much water flooding the house... So we all slept in the wet... all together on the floor”. Group 3 - Young people from the periphery

“...we can only rely on each other, the community....”

______________________________________________________________________

## 3.7 Change in future plans associated with CC

“*I was going to buy a house, but now I can’t anymore because I have to fix things here. I lived through a flood and lost all my furniture.*”. - Group 2 - Women community leaders

“*It’s not that big, but my dream since I was a child was, like, I've always loved landscaping, gardening, to spend my retirement at my grandfather’s place. And I know there won’t be any water by the time I get there, so I let it go. I took my dreams away from there...”-* Group 6 - Young university students

## 3.8 Involvement in individual and collective activities to combat CC

“*And we have to have time for ourselves, we work, that’s it, we work like sons of bitches, 16 hours a day, 18 hours a day. It’s how it is, it’s not just that we don’t collect rubbish, we can’t take care of a lot of things.”* - Group 2 - Women community leaders

“*I even wrote a poem about it called ‘The world is not ours’. And it’s a poem that I really like because many people are outraged, but there’s no point in being outraged if your outrage goes unanswered. There’s no point in seeing someone throw a piece of paper on the ground if you don’t do anything about it*.” - Group 3 - Young people from the periphery

“*I'm affiliated to a lot of parties, not political parties, but parties that are concerned about the environment. I’m affiliated to the UMES party, which is the Municipal Union of Secondary School Students. I like to be actively involved.*” – Group 5 - Young people from the periphery

“*But there are certain actions that we can carry out as individuals that will make a difference, even if it’s minimal. One example, for me, is diet. So, like, you don’t have to go vegan, you don’t have to go vegetarian, but one day a week when you could eat less meat, ... There are studies by NGOs showing how much effect this has. It doesn’t seem like much, but a year like that, imagine the rest of your life? That makes a big difference*.” - Group 6 - Young university students

## 3.9 The role of the State, institutions and other agencies

“*Because we know who’s responsible for this...... man is responsible for climate change, so the state is also responsible for climate change, and it’s also responsible for providing for these families because of the disasters they’ve caused. Because, for example, there in Vaz de Lima, that watercourse never flooded, but then Sabesp came to Vaz de Lima to do the water supply and they promised, like, ‘you’re going to have a steady supply’ blah blah blah, and the work they did wiped out the neighbourhood because the water started rising as soon as the work began. The work was finished, and the water started to rise. So I’m thinking a lot about this revolt against the State, which destroys, but also can’t compensate for what it itself caused and we’re left there defenceless (...) Anyway, no one is responsible, right, for these climatic disasters (…)”* - Group 2 - Women community leaders

“*I’m not going to say that I don’t have my own opinion on this, because the feelings that all these people have make me angry. Because I know that the State, the government, has the resources for this, it has the resources to teach people how to act right, to respect both ourselves, as human beings, and nature itself, because that’s where our food comes from. That’s where everything we have today comes from, especially our homes*.”- Group 3 - Young people from the periphery
