# Supplementary material for: “No forest, no future, but they don’t see us”: eco-anxiety, inequality, and environmental injustice in São Paulo
Source: Front Public Health. 2025 Jun 5;13:1555386. doi: 10.3389/fpubh.2025.1555386 (PMC12176893; doi:10.3389/fpubh.2025.1555386)
Supplement: Supplementary file 8 [file Data_Sheet_8.docx]

**Annex G.** Synthesis of the key findings.

| **Category** | **Key Findings** | **Population/Comments** |
| --- | --- | --- |
| Perception of Climate Change | Understanding of climate change varies: in vulnerable communities it is linked to direct experiences and extreme weather events, while university students understand it through formal education and digital media. | Vulnerable communities vs. university students. |
| Feelings and Emotions | Vulnerable communities express intense feelings of sadness, fear, hopelessness, and trauma; in contrast, university students report anxiety, resignation, and frustration regarding distant events. | Higher emotional intensity in vulnerable groups. |
| Concrete Experiences | Direct experiences include personal losses and damage to housing in vulnerable areas; students generally describe less personally impactful events. | Examples include loss of family members and deterioration of living conditions. |
| Social Inequalities | There is a marked perception of environmental injustice: marginalized populations disproportionately suffer from the impacts of climate change, highlighting the link between poverty and vulnerability. | Criticism of economic elites and the lack of attention to the disadvantaged. |
| Health Impact | In vulnerable communities, there is an increase in physical health problems (allergies, infections, fatigue) and mental health issues (panic attacks, PTSD, depression), while students mainly mention anxiety. | Compromised health in areas with higher socioeconomic vulnerability. |
| Support Networks | Family and community networks are vital for coping with climate crises; however, there is insufficient access to mental health services through the public system. | Importance of social support in the face of inadequate institutional response. |
| Future Plans | Material and health impacts in vulnerable communities alter their future plans (e.g., delaying studies or making other life adjustments), whereas students make long-term decisions such as postponing parenthood. | Life planning changes based on exposure to extreme climate events. |
| Pro-environmental Activities | Individual sustainable behaviors (recycling, water and energy conservation) are practiced, although collective action is limited, partly due to economic constraints. | The ability to adopt sustainable behaviors is conditioned by the socioeconomic context. |
| Role of the State and Institutions | Government inaction and the prioritization of economic interests over environmental protection are criticized, which exacerbates the vulnerability of marginalized communities. | Lack of policies and resources in environmental education, infrastructure, and inequality reduction. |

PTSD= Post Traumatic Stress Disorder
